# The benefits of role play in the development of drawing in preschool children

**DOI:** 10.3389/fpsyg.2022.1010512

**Published:** 2022-11-24

**Authors:** María del Rosario Bonilla-Sánchez, Marco Antonio García-Flores, Ignacio Méndez-Balbuena, Jocelyn Guadalupe Silva-González, Ernesto Vladimir Ramírez-Arroyo

**Affiliations:** Facultad de Psicología, Benemérita Universidad Autónoma de Puebla, Puebla, México

**Keywords:** preschool, role play, symbolic function, drawing, children development

## Abstract

**Objective:**

The purpose of our study was to demonstrate the benefits of role play based on a Historical–Cultural perspective with the implementation of symbolic elements generated by the development of drawing of preschool children from suburban origin in a group of normal Mexican preschool children. We predict that the quality of the drawings will be improved after the preschoolers participate in such a role play program.

**Methods:**

A pre-experimental study was carried out, with pre-test and post-test assessments. The drawings were assessed with quantitative (frequencies of the quality of the parameters) and qualitative (characteristics of the children's drawings) methods. The role play program was based on Elkonin's method, activity theory, and P. Ya Galperin's formation by stages of mental actions. The program encouraged the use of graphic signs and symbols, with the intention of representing situations, actions, objects, and/or contents. Twenty-six preschool children participated in our study.

**Results:**

To know the effect of the role play program with symbolic elements on the drawing of preschool children, we performed a quantitative and qualitative analysis, a Wilcoxon test contrast between the initial and final assessment. Both analyses showed a significant increase in the scores in all the tasks with a medium–large effect size, being the largest “Pictograms” and “Drawing of the route”; and in the analysis of the qualitative parameters, specifically in “Maintains the objective,” a significant increase was found in all the tasks.

**Discussion:**

Role play, being the main activity in preschool age, develops all aspects of the child's psychic life. The importance of role play in a preschool child makes it possible to work in the zone of proximal development with the regulatory function of language, with the planning, execution, and control of playful activity and, most importantly, with the use of various materials and perceptual-graphics tools. These findings showed an improvement in the development and complexity of the symbolic function in the drawings.

**Conclusion:**

Role play, as a work methodology in preschool age, leads to the use of new meaning systems that prepare the child to carry out present and future complex symbolic actions.

## Introduction

Child psychological development is a continuous process where new formations arise that determine the psychological and social changes at each age, determining the most important aspect of the child's consciousness and his relationship with the environment (Vigotsky, 1995a). From the point of view of Historical–Cultural psychology, there is a particular form of activity of the child during early childhood (preschool age) that is the true condition that transforms psychological development (Leontiev, 1984; Talizina, 2009).

Child psychological development theorists have pointed out that in preschool age, role play is the activity that promotes the development of all psychological processes such as the acquisition of affective–emotional relationships and existing roles in society, setting the platform for the formation of the child's personality, as well as the new psychological formations corresponding to this age such as symbolic function, imagination, orientation in human relationships and actions with a conscious character, as well as thoughtfulness and voluntary activity (Liublinskaia, 1971; Elkonin, 2010). In this way, role play is a simulation of the social interactions interpreted by the children's limited view of the world, which will be expanded with the aid of other children or an educator who highlights the most important aspects of human activity.

Within the formative stages of role play, we find symbolic play which guarantees that the child's actions are generalized by substituting one object for another according to the game's theme (Talizina, 2009; Solovieva and Quintanar, 2012). Thus, symbolic play drives and turns more complex the development of the symbolic function. It is also a psychological formation that characterizes children's development in preschool age, which allows them to explain representations and schematizations of actions and concrete objects with some type of symbolic representation, such as gestures, images, or words (Salmina, 2010; Ruíz and Abad, 2011). The symbolic function reflects the level of mastery of psychological tools, specifically of signs and symbols, as instruments that qualitatively modify the psychic life of the human being (Vigotsky, 1996; Bonilla-Sánchez and Solovieva, 2016). The symbolic function is an essential component for the formation of mental images. The child's action leads at the same time to the formation of the image of the action itself, and the characteristics of that object image (Galperin, 2009). This function allows the child to evoke mental images by representing realities not present in their drawings.

Cognitive psychology points out that in early childhood, the child will be able to represent and transmit information through symbols, gestures, drawings, games, etc., and will be able to solve practical problems, plan the use of simple tools, and expand their practical knowledge, all these characteristics coming under the scope of sensorimotor intelligence (Piaget, 1984; Saldarriaga-Zambrano et al., 2016). However, this process is not innate and requires an educational process through the systematic introduction of children to social roles and activities.

It is important to mention that sensorimotor actions are carried out slowly and consecutively, while representational symbolic thought can occur faster, since it covers the past, present, and future events. Therefore, sensorimotor intelligence is oriented to concrete and immediate actions, on the other hand, symbolic thought is oriented to knowledge, actions, and consequences provided by objects and their generalization (Flavell, 2019).

During play, the ludic use of objects leads the child to use the socially established symbolic system, making a gradual use of various tools, such as drawing and language. For the child, the word that designates the object contains a system of actions, as well as the peculiarity of the object or the phenomenon to which the word itself refers, this aspect of language fulfills a generalizing function. Moreover, during play the language is used as a regulatory tool of the child's behavior. First, the adult regulates it with her/his language, then the child controls himself with his external language and finally controls himself with his internal language (Luria, 1984; González et al., 2011; Rabello, 2014). This control is present during play in its organization, execution, and verification (García et al., 2013). This leads to the minor developing reflexive thinking, which manifests itself when the child manages to transform his own verbal expression and actions (González et al., 2011). On the other hand, drawing requires the substitution of the essential and distinctive characteristics of objects and situations. It is a symbolic perceptive action of the practical activity of the child (Rojas, 2012; González-Moreno and Solovieva, 2016). This is how the execution of perceptual actions or drawings maintains a close relationship with the conformation of the internal images of objects.

In addition, drawing constitutes a form of representation of reality (Furth, 2002), beginning in the plane of perceptive actions that, initially were concrete, which later will originate internal images of objects to finally become abstract ideas and concepts in the plane of mental actions (Galperin, 2009).

When the children can understand that they can also express ideas, feelings, a communicative intention, and meanings in drawings, these will constitute forms of symbolic representation created by them in advance, planned, organized, and thoughtful (Vigotsky, 1995b; Salsa, 2004). For this reason, it is of great importance that children know, understand, and use various types of symbolic tools from an early age (DeLoach, 2011). Such tools appear during role play as substitutes for real objects to aid the children carry out the ludic situation.

The importance of the development of internal images through drawing is such that previous studies have indicated that a poor development of internal images would be a negative indicator in child development that will affect the learning of oral and written language (Akhutina and Zolotariova, 2001; Solovieva et al., 2002; Rojas, 2012; Solovieva and Quintanar, 2012).

This is how the role play is the quintessential activity to give way to the development of the mental actions of children in preschool age, from the external material plane to the internal mental plane, actions formed in the activity of the child and his interaction with objects (Elkonin, 2010).

However, practical actions and use of signs can operate independently in young children, therefore symbolic activity is the way by which the adult guides the child to produce novel forms of behavior using external and internal psychological tools (Salmina, 1988; Vigotsky, 1995a). During play, children need an adult to tell them how adult people behave and how can they represent such behavior, what actions are important to be played, and what objects are needed to perform such actions.

It has been shown that it is possible to modify the type of response of children guided by adults during memory and attention tasks, using external tools or signs, such as cards, colors, images, etc. The children who paired these tools with information were the ones who were able to remember it more easily, that is, children used the mediatization of answers (Vigotsky, 1996). Other studies confirm that with the use of external signs, children were able to solve problems or conflict situations within the role play activity. For example, children were able to pair a graphic symbol (drawing) with a meaning both to substitute real objects and to add content during role play (Bonilla-Sánchez and Solovieva, 2016; Bonilla-Sánchez et al., 2019).

The development of the symbolic function in preschool age implies the possibility that children dominate the use of signs and symbols with intention and meaning according to their age and sociocultural environment. It is a psychological training, to prepare the child for admission to systematized education in primary school, where the child now must operate with more complex tools, such as the alphabetic and numerical systems.

Therefore, a human being right from childhood has that great capacity not only to process symbols but also to process object-specific information. That is, individuals are capable of computing conceptual information, as cognitive psychology points out. Hence, the importance of considering how infants process attention, what motivations they have, how they encode information, how they relate it to previous knowledge, how they store new knowledge, and how they retrieve it (Shuell, 1986) is paramount, so that there is significant learning, that is, lasting internal changes observed in intellectual abilities, verbal information, cognitive strategies, motor skills, and attitudes (Gagné, 1985; Eysenck and Keane, 1994). Considering these aspects represents great educational challenges. The purpose of our study was to demonstrate the benefits of role play with the implementation of symbolic elements generated by the development of drawing of preschool children from suburban origin. We predict that the quality of the drawings will improve after preschoolers participate in a role play program.

## Materials and methods

### Participants

Twenty six healthy participants (16 girls and 10 boys, mean age 5.5 years) without any history of a neurological disease took part in this study as a natural group (Campbell and Stanley, 1982). A local Ethics Committee approved the experimental protocol. All children participated according to the declaration of Helsinki (Asociación Médica Mundial, 2013).

The level of education of the parents, of the participating children, was elementary school and incomplete middle school; the mothers of the minors worked as seamstresses, domestic workers, and those doing housework, while the fathers worked as construction workers, drivers, or artisans. The socioeconomic status of the participating children's community was low.

### Experimental design

A pre-experimental study was carried out, with pre-test and post-test assessments. The independent variable was the implementation of a role play program of symbolic elements generated by the development of preschool children; the dependent variable was the quality of the drawings. The drawings were assessed with quantitative (frequencies of the quality of the parameters) and qualitative (characteristics of the children's drawings) methods.

The inclusion criteria were the participants had to be between 5 and 6 years of age and should have attended the third grade of preschool education in an initial educational institution incorporated into the Public Education System (SEP), in Tlaxcala, México. The exclusion criteria were the participants above 6 years of age, had not regularly attended the activities of the game program, and had neurodevelopmental disorders or specific learning difficulties.

### Assessment instrument

To analyze the drawings of the participants in the pre-test and post-test assessments, we used the tasks corresponding to symbolic perceptual actions (Table 1) of the Symbolic Function Evaluation Protocol (Solovieva and Quintanar, 2014). This instrument was designed based on the Historical–Cultural Child Psychological Development Model (Vigotsky, 1995a, 1996). The evaluators asked questions to the children to get them explain their drawings and the evaluators recorded their actions and expressions while the children drew.

**Table 1:** Tasks applied for the assessment of symbolic perceptual actions.

**Tasks**	**Instruction for the child**	**Help given to the child**
1. Pictograms. Words: sickness, happy party, development, patience.	“*Make a drawing that matches the following words”* When the children finished the drawing he is asked the following: • What did you draw? • Why did you draw that?	• Repeat the words. • Verbal motivation for the child.
2. Prepare a letter.	“*Without words and only drawings, prepare a letter for your mom (dad) about what would you like to eat on Sunday.”* • What did you draw? • Can you explain the drawing?	• Repeat the instruction. • Motivate the child to produce the drawing. Guide questions: •What do you like to eat? • Draw your favorite meal.
3. Draw the route (path)	“*Draw the route (path) from your home to the nearest store*” • What did you draw? • Where you come from and where are you going? • How do you know where the route begins and ends?	• Repeat the instruction. • Motivate the child to produce the drawing. Guide question: •What places are near your home?
4. Drawing places in the city	“*Draw in this sheet of paper the places of the city”* • What did you draw? • Can you explain the drawing?	• Repeat the instruction. • Motivate the child to produce the drawing. Guide question: •Which place you like the most?

### Procedure

After receiving prior authorization from the authorities of the preschool institution, the objectives, scope, and schedule of the research were presented to the parents of the children to obtain their consent to make the children participate. Subsequently, the initial assessment was applied individually. The evaluator gave instructions (and if necessary, even provided help) while the child drew (Table 1). Later, the child was asked to explain his drawing, and the child's verbal account was recorded. The tasks were administered by Neuropsychology specialists, in work cubicles. The experimental session was individual and lasted ~30 min, during which participants sat comfortably in a well-ventilated and illuminated room.

Afterwards, a role play program with symbolic elements was applied 4 days a week at the specific time fixed by the group's main educator, for a period of 5 months. The game sessions lasted 1 h. A final drawing assessment was applied using the above-mentioned tasks. Qualitative and quantitative analyses were made of the drawings to compare the pre- and post-assessments.

### The role play program

The aim of the role play program was to apply the role play stages (Elkonin, 1980), also considering the stages of development of the symbolic function (Salmina and Filimonova, 2001), the theory of activity (Talizina, 2009), and the formation by stages of mental actions (Galperin, 2009). The main objective of the program was to encourage children to use graphic signs and symbols, with the intention of representing situations, objects, and/or contents, during the role play scenarios, thus contributing to the formation of the symbolic function, as an intrinsic psychological function of the preschool age.

## Data analysis

### Qualitative analysis of the drawings

We analyzed the characteristics of children's drawings based on the categories and parameters described in Table 2, in the four tasks evaluated.

**Table 2:** Categories and parameters for the qualitative analysis of the drawings.

**Categories**	**Parameters**	**Description**
Execution of traces	Requested drawing. Unrecognizable scribble.	The child produces the requested drawing. The child recognices and names his drawing with out losing the objective. Repetitive and disorganized traces. The draw cannot be interpreted by others.
Intention or reason to represent the idea or content	Keeps the objective. Loses the objective.	The drawing matches the task's objective, and the children can explain it. The drawing does not match the task's objective.
Correspondence of the drawing	Presence of graphic elements in relation to the objective. Simple drawing.	The drawing includes essential details and elements, the children can explain them according to the task's objective. The drawing lacks essential details and elements, they are simplified, and the child's verbal expression does not match the task's objective.
Structure of the drawing	Keeps the structure of the drawing.	The drawing is organized on the sheet of paper, good proportion and distribution of the traces.
	Does not keep the structure of the drawing.	The drawing is not organized on the sheet of paper, the proportion is not right, and the traces are unevenly distributed.
Verbal report of the drawing	Use of clear and complete sentences.	The child explains his drawing with sentences related to the task's objective with subject and predicate.
	Use of verbs.	The child includes verbs to explain his drawings.
	Use of prepositions.	The child uses some prepositions for explaining his drawing (to, for, toward).
	Use of nouns.	The child explains his drawings using only nouns.
	Reduced verbal expression.	The child does verbal expressions using some nouns unrelated to the task's objective. The child does not use verbal expressions about the drawing.

### Quantitative analysis

We measured the presence and frequency of qualitative parameters in the four tasks evaluated. According to the performance of the children in the assessment, we assigned the value 3, when child made the drawing adequately, according to the stipulated parameters of the expected drawing, and independently, the value 2, when the child drew adequately, but with the help of the evaluator, and the value 1, when the child did not make the expected drawing, despite the help provided.

### Statistical analysis

To know the effect of the role play program with symbolic elements on the drawing of preschool children, we performed a quantitative analysis, serving as a contrast between the initial and final assessment. Because our data were not lognormally distributed (Kolmogorov–Smirnov normality test, *p* < 0.05), we used a non-parametric repeated-measures (Wilcoxon) test, under the null hypothesis that the dependent variables were the same in both conditions (initial and final). The analyses were performed in the Statistical Package for the Social Sciences (SPSS) version 25. The test was one-tailed for calculating the statistical significance. All effects are reported as significant for *P* < 0.05.

## Results

Table 3 shows the results of the Wilcoxon test to contrast the qualitative parameters obtained from the drawing tasks of the assessment during the initial and final assessments. The mean, standard deviation (SD), the *Z* statistic, the statistical significance *p*, and the effect size *r* are also shown in Table 3. A statistically significant difference was found in all the variables, favoring the final condition over the initial one. For the quantitative analysis, we found that the effect size was medium–large for all variables, with an average of 0.45 for all tasks.

**Table 3:** Statistical results for quantitative analysis.

	**Initial assessment**		**Final assessment**		**Wilcoxon test**		
**Task**	**Mean**	**SD**	**Mean**	**SD**	***Z***	***p***	***R***
Pictograms	1.48	0.40	2.0	0.25	−3.97	0.0001	0.55
Prepare a letter	2.54	0.76	3.0	0.19	−2.42	0.05	0.34
Draw the route (path)	2.0	0.89	2.85	0.46	−3.51	0.0001	0.49
Drawing places in the city	1.61	0.85	2.5	0.86	−3.09	0.005	0.43

**Figure 1:**
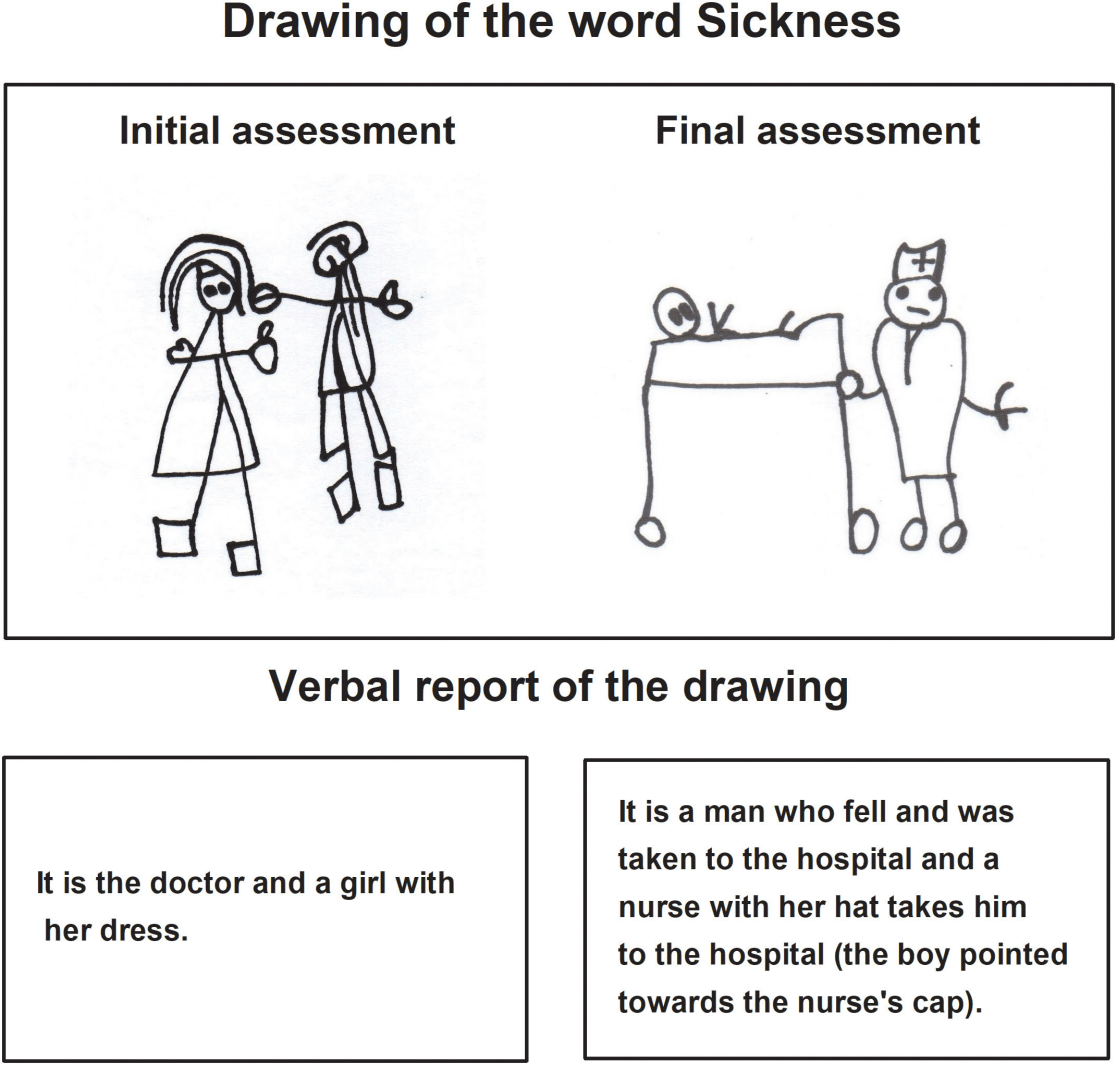
Child A drawing in the pictogram task during the initial and final assessments.

**Figure 2:**
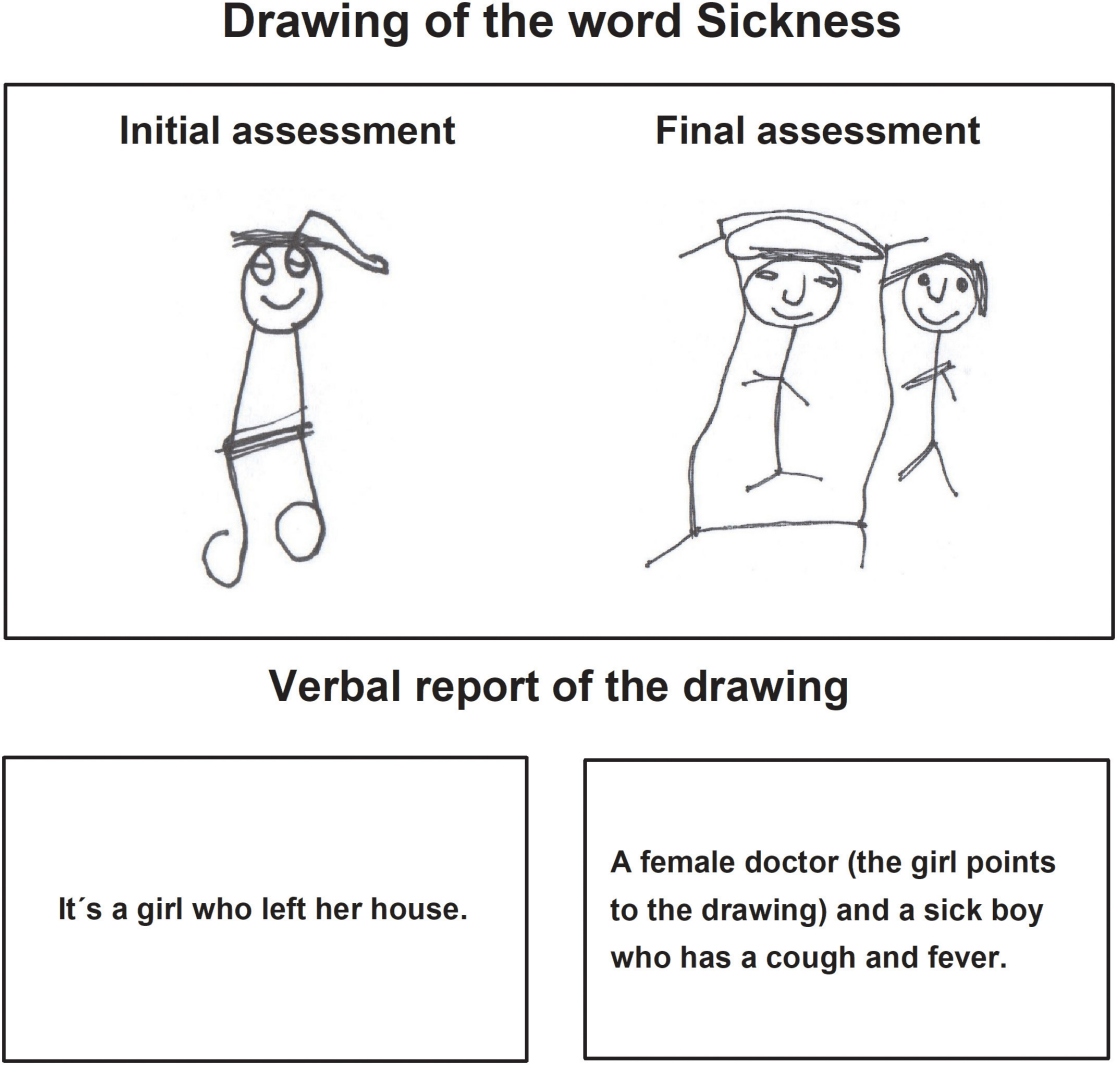
Child B drawing in the pictogram task during the initial and final assessments.

Regarding the *Pictograms* task, the main qualitative characteristic of the children's drawings in the final assessment pertained to the fact that they made the expected drawing, according to the evaluator's instructions (Figures 1, 2). The children recognized and named their drawing, explaining it without losing the objective. The traces included essential elements and details to identify images, qualitative characteristics that were not present in the drawings of the initial evaluation. Drawing was now organized and proportioned in graphic space.

The verbal report of the children about their drawings of the final assessment expressed complete sentences integrating mainly nouns and verbs. The stories were related to the instructions indicated by the evaluator.

**Figure 3:**
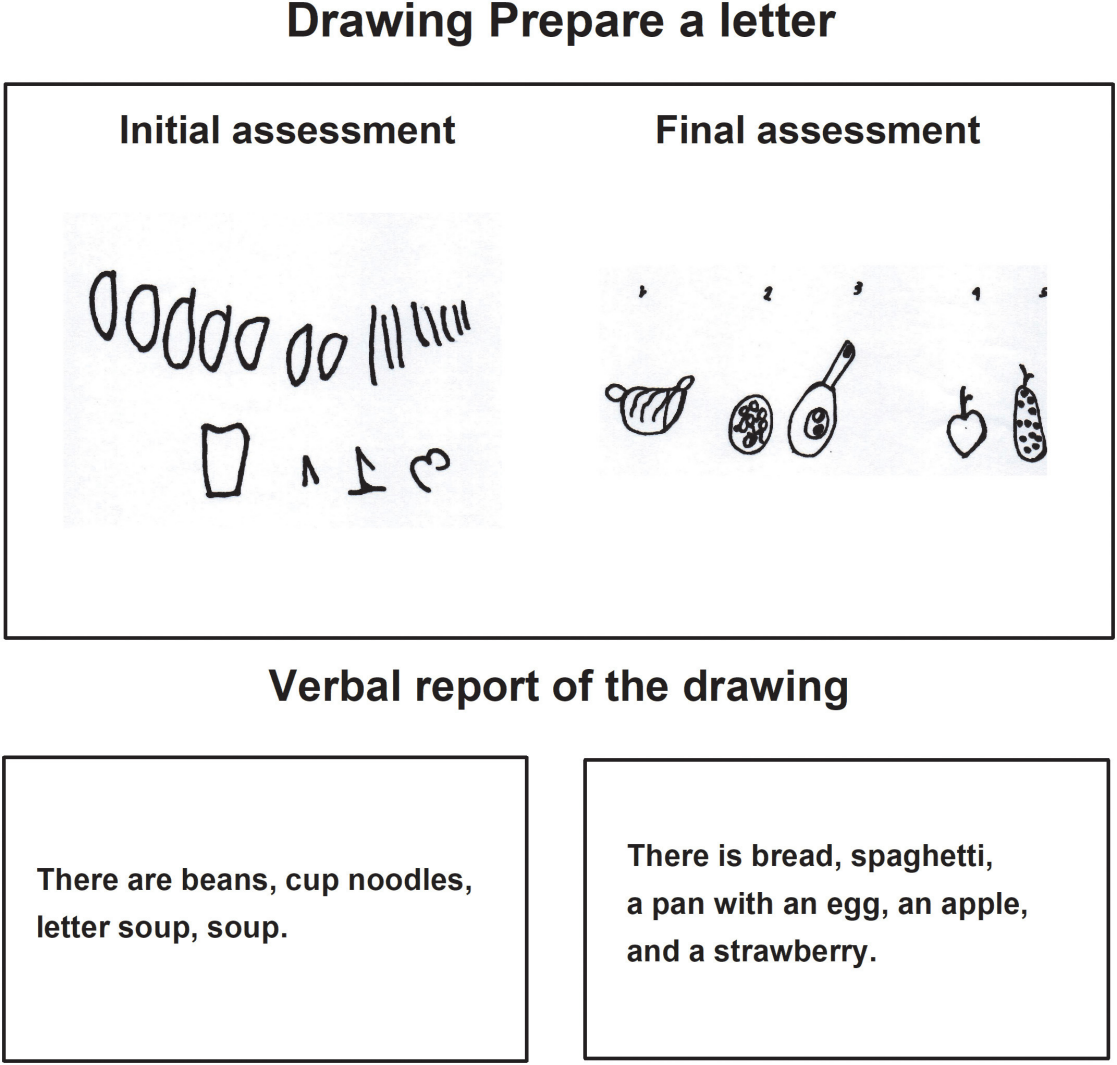
Child C drawing of the task prepare a letter during the initial and final assessments.

**Figure 4:**
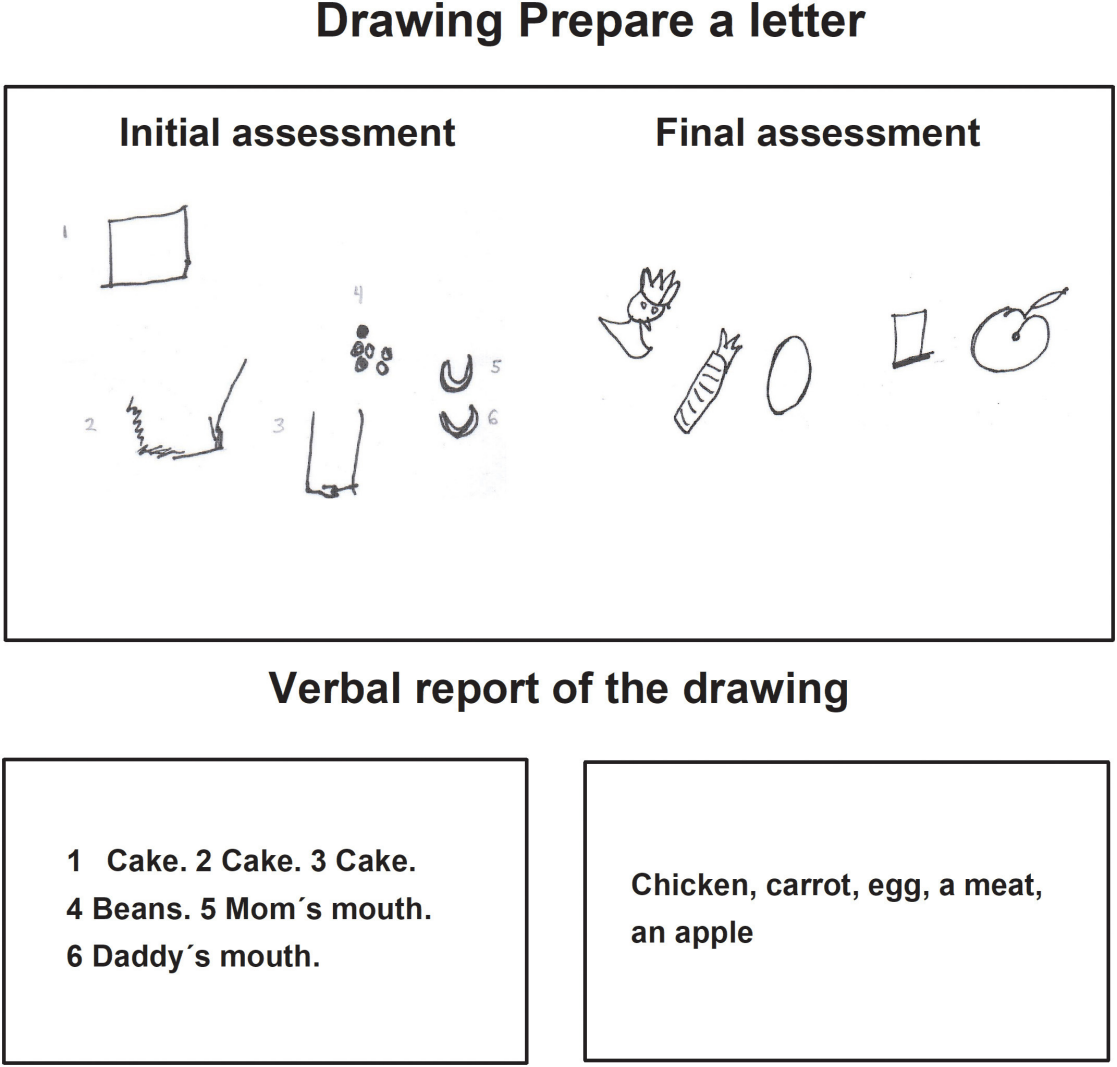
Child D drawing of the task prepare a letter, during the initial and final assessments.

**Figure 5:**
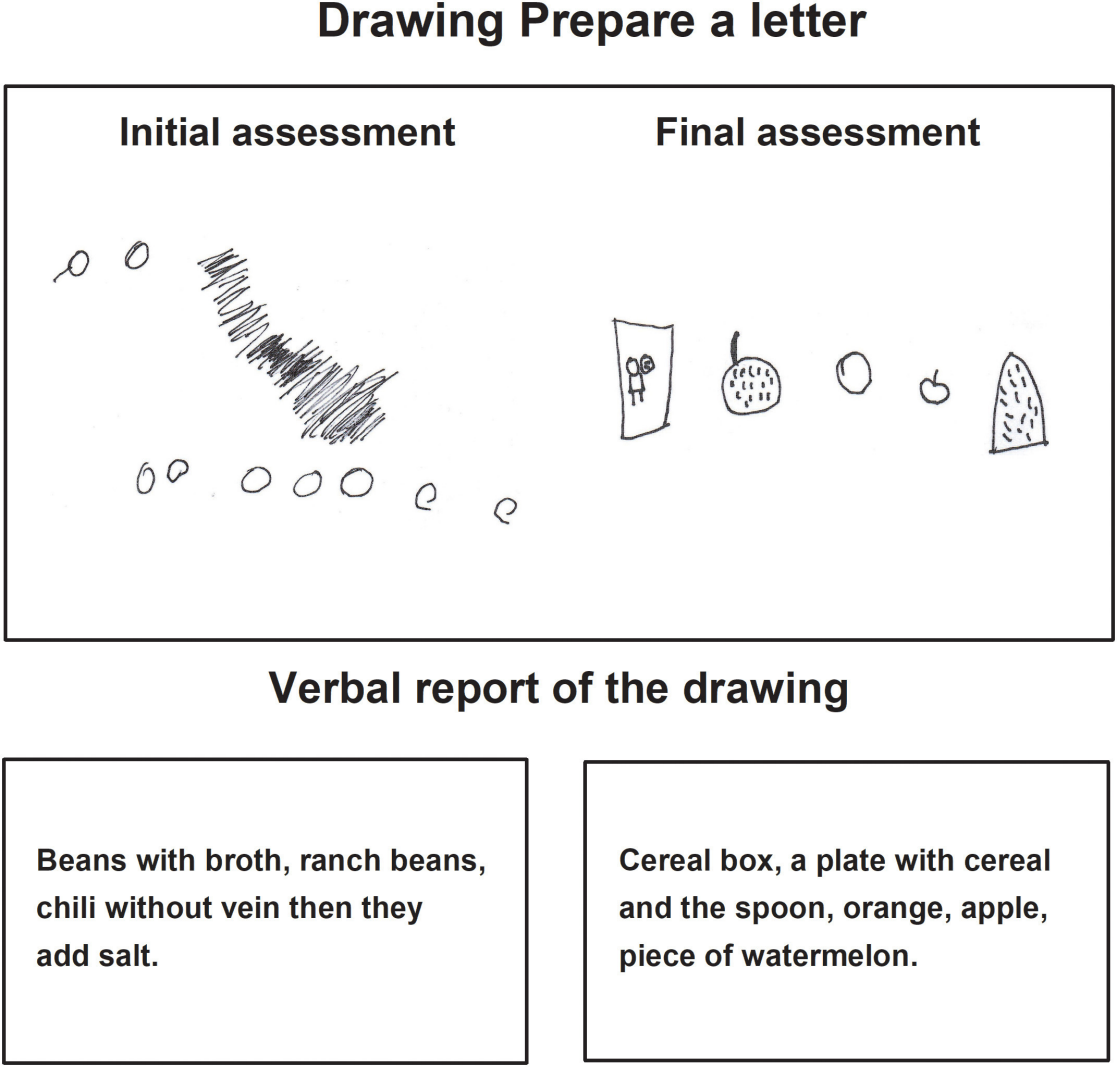
Child E drawing of the task prepare a letter, during the initial and final assessments.

In the task *Prepare a letter* in the final assessment, children made drawings where the motive to represent an idea or content can be observed, the drawings contained details in correspondence with the image and were identified and explained by the child thoroughly. Those characteristics were not present in the initial drawing, in which, the drawings were repetitive and without details of the images they represented (Figures 3–5).

In the verbal report, during the final assessment, the children showed their drawings to the evaluator, pointing to each one of them while they named them sequentially from left to right (Figure 3).

**Figure 6:**
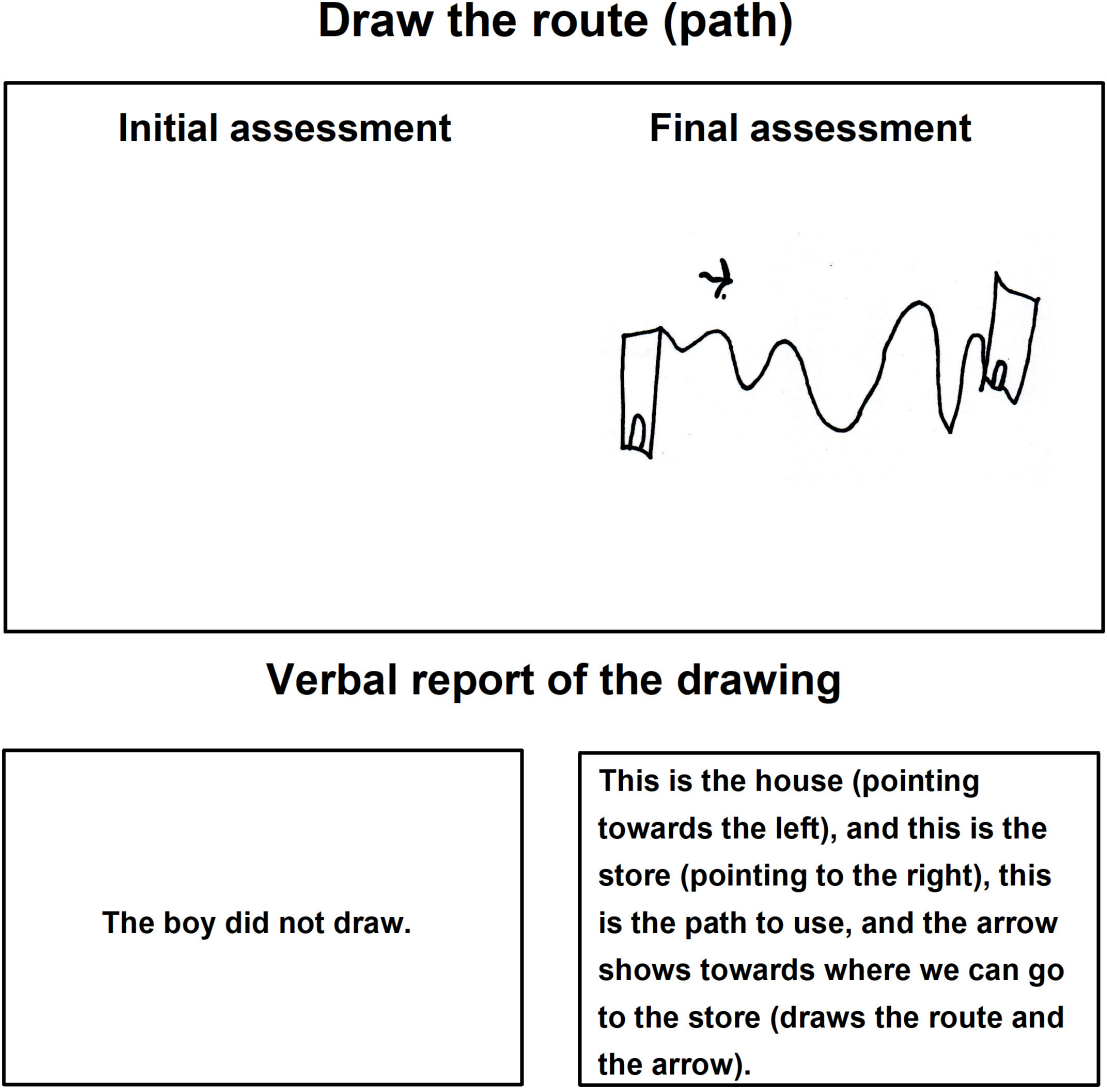
Child F task drawing the route (path) during the initial and final assessments.

**Figure 7:**
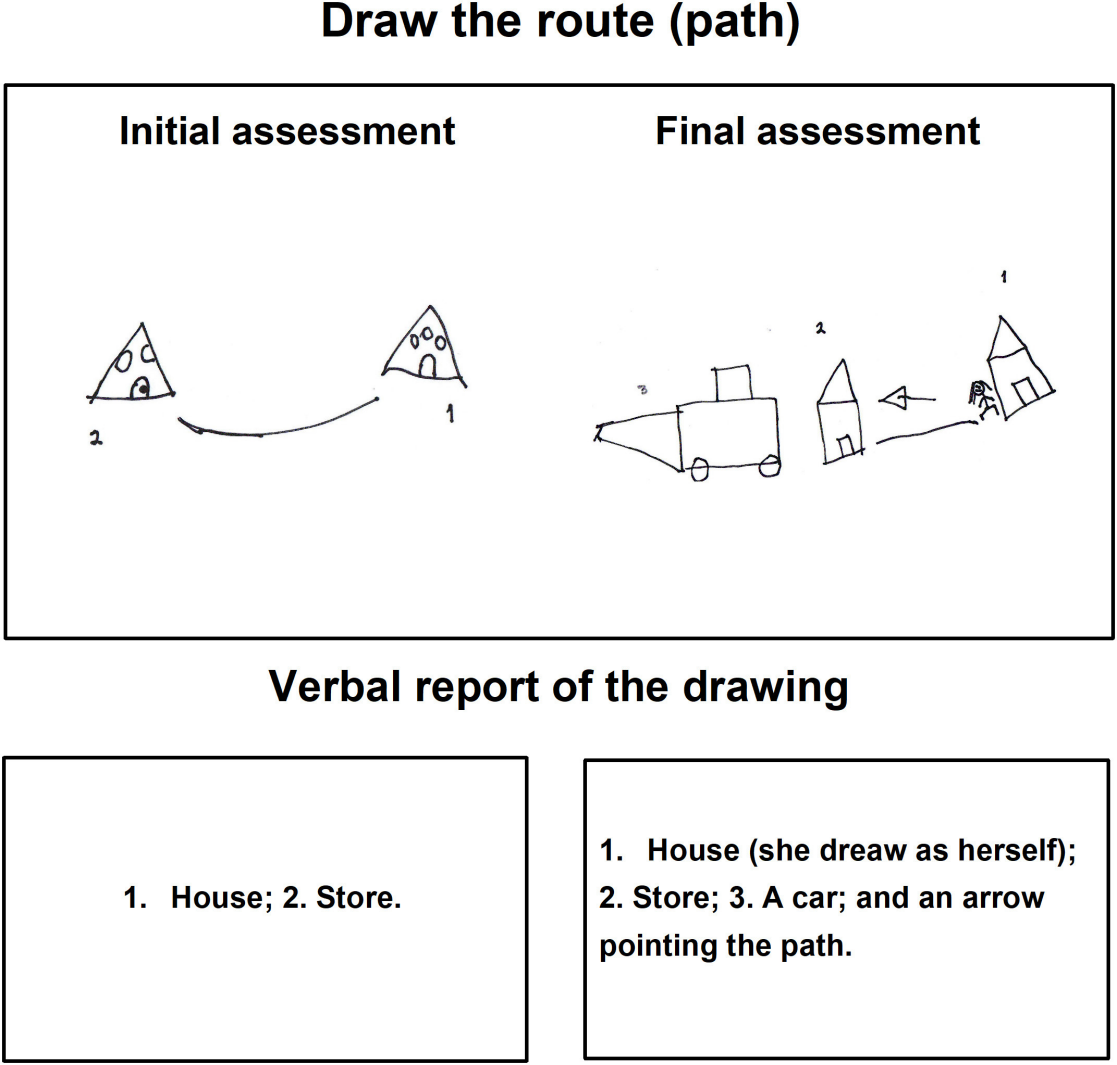
Child G task drawing the route (path) during the initial and final assessments.

On the other hand, during the task *Draw the route (path)* in the initial assessment, some children drew the house or the store, but they could not represent the route. Figures 6, 7 show, a child who did not produce the drawing according to the instruction, however, the very same child in the final assessment made the expected drawing, organized his drawing in proportion to the space on the sheet including the arrow sign to indicate the direction of the route toward an objective in a way reflective.

In the verbal report (Figure 6), the child explained his drawing by expressing complete sentences with nouns and prepositions, at the same time pointing out the elements of his drawing while showing them to the evaluator.

**Figure 8:**
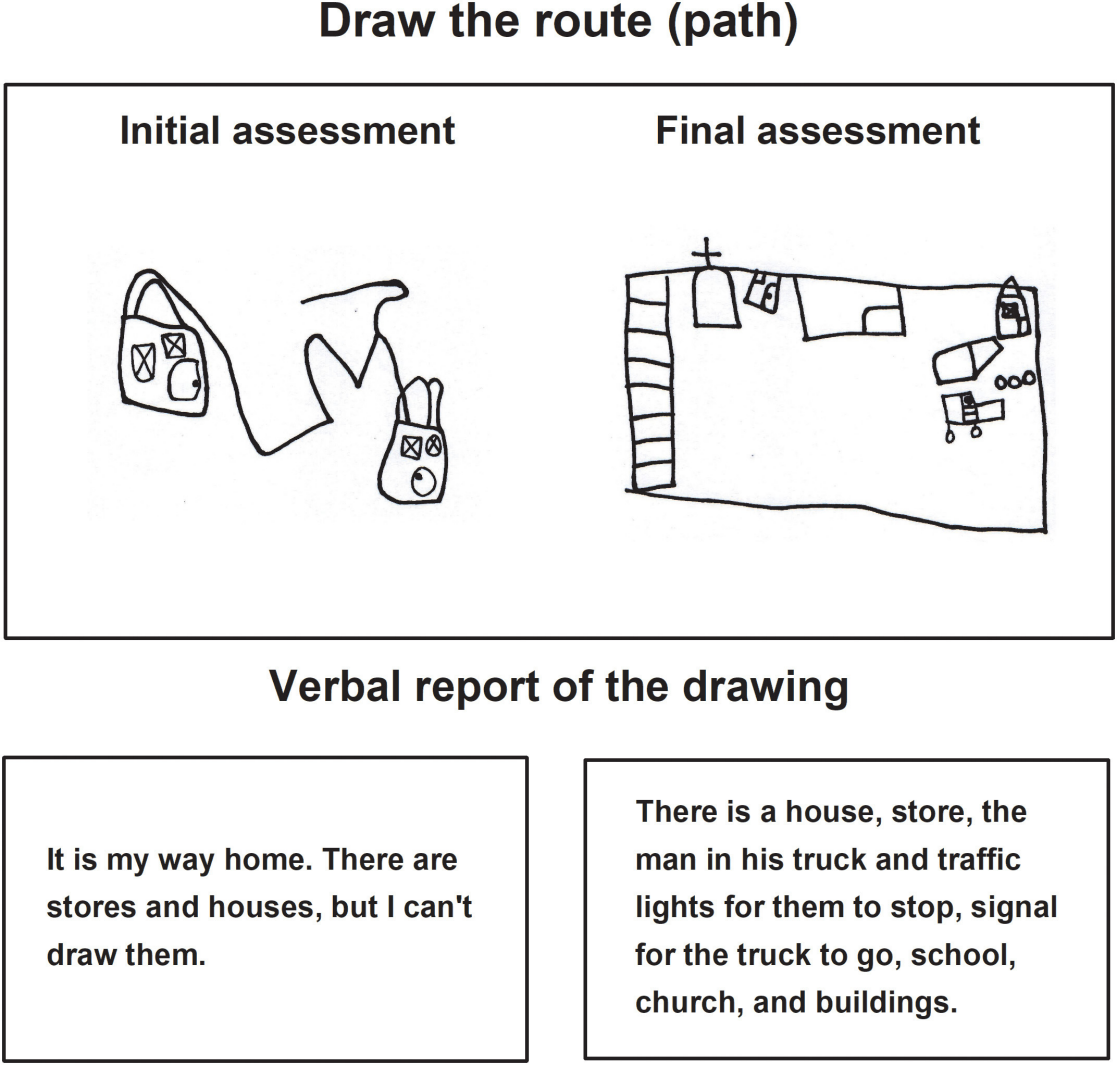
Child H drawing of places in the city during the initial and final assessments.

**Figure 9:**
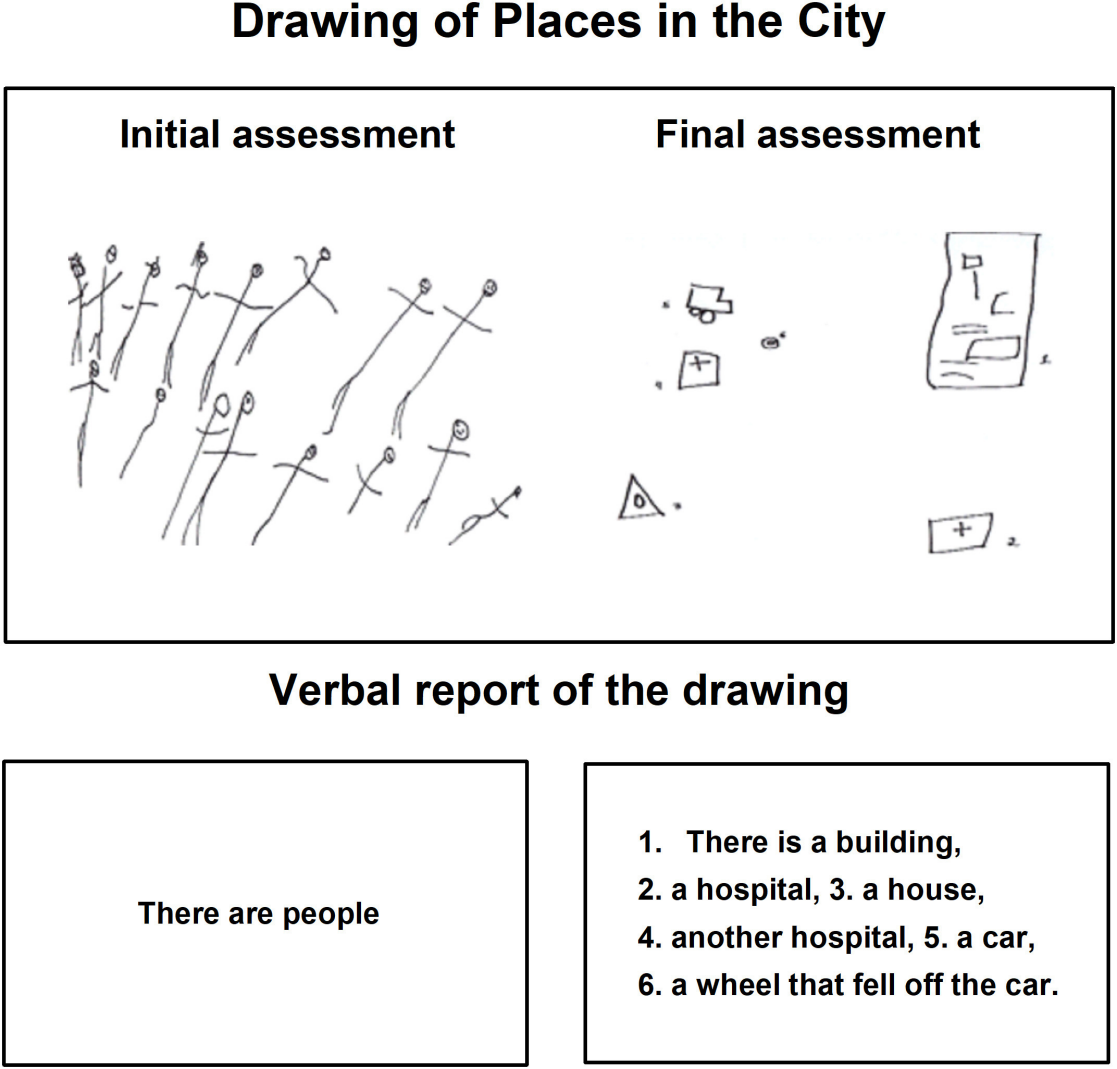
Child I drawing of places in the city during the initial and final assessments.

In the task *Drawing Places in the City* (Figures 8, 9), during the initial assessment the drawings did not match the instruction and various objects and/or details did not appear. Some drawings were even repetitive, such as that drawn in Figure 8, where the child continued to make the route from his house to the store, executing the drawing corresponding to the instruction of the previous task.

The repetitiveness was also present in the verbal account since the minor continued explaining the drawing of the route from his house to the nearest store. However, during the final assessment the same child represented various objects with minimal details, but was able to name them, point them, and explain his drawing to the evaluator.

Finally, regarding categories and parameters for the qualitative analysis of the drawings (Table 2), one category which is of particular interest is the category *Intention or reason to represent the idea or content* in the drawing. The results showed that during the initial assessment, the majority of children did not maintain the objective of the task, as observed in the drawings in Figure 4, but in the final assessment they managed to execute the expected drawing. The findings of the qualitative differences are in line with the results of the significant differences between the initial and final assessments for the quantitative variables of the parameter *Maintains the objective* in each of the tasks (Table 4).

**Table 4:** Statistical results of qualitative analysis for the parameter Maintains the objective in the tasks.

	**Initial assessment**		**Final assessment**		**Wilcoxon test**		
**Task**	**Mean**	**SD**	**Mean**	**SD**	***Z***	***p***	***R***
**Pictograms**
Sickness	0.46	0.51	0.73	0.45	−2.646	*P* < 0.01	−0.37
Happy party	0.42	0.50	0.61	0.49	−2.236	*P* < 0.05	−0.31
Development	0.46	0.51	0.73	0.45	−2.646	*P* < 0.01	−0.37
Patience	0.46	0.51	0.73	0.45	−2.646	*P* < 0.01	−0.37
Prepare a letter	0.50	0.51	0.62	0.49	−1.732	*P* < 0.05	−0.24
Draw the route (path)	0.27	0.45	0.69	0.47	−3.051	*P* < 0.001	−0.42
Drawing places in the city	0.38	0.50	0.65	0.48	−2.333	*P* < 0.01	−0.32

## Discussion

Drawing in preschool age is a symbolic perceptive action that reveals the practical activity of the child and his social environment, and it is a form of symbolic representation of the child's reality and shows how internal images of the external world are being formed.

The purpose of our study was to demonstrate the effect of a social role play program with symbolic elements over the enhancement of the characteristics of the drawings of preschool children. Analysis of the effect sizes of the drawing program on the quality of the drawings showed that they were medium–large. For the quantitative analysis, we reported a medium–large average effect size of 0.45. In particular, the pictogram tasks had an effect size of 0.55. According to Cohen (1992), values above *r* = 0.5 are considered large, which adds to the robustness of the applied method.

The qualitative analysis showed that, according to the expected categories and parameters (Table 2), initially the children made drawings without the characteristics related to the instruction, even with the help provided by the evaluators. In the final assessment, the children obtained the value 3, the highest for making the expected drawings without receiving any type of help, results that coincide with previous investigations that refer to the fact that the game leads to the development of the symbolic function, promoting the capacity of the children to use objects, actions, words, and people to represent something different, which prepares the child for the acquisition of drawing, reading, and writing skills (Salsa, 2004; Salmina, 2010).

The role play is confirmed as the leading activity of psychological development in preschool age, producing new psychological aspects (neoformations): the symbolic function, the imagination, the orientation in human relationships and actions, the conscious orientation in these, as well as thoughtfulness and voluntary activity (Elkonin, 1980; Davidov, 1988; Vigotsky, 1995a,b,c; Bodrova and Leong, 2003).

With the implementation of graphic activities during ludic activity, children learn how to use symbols as substitutes by understanding the purpose of the object during a particular social activity. For example, circles can only be used as money when buying things, but the same circles can represent vegetables in a market. They will even use symbols different from those used in other games, discovering that they can represent an object, a situation, a communicative intention, ideas, and feelings, actively ordering their reality and shaping specific purposes (Cox S., 2005).

In this way, the drawing will constitute a planned, organized, and thoughtful symbolic representation, where the children include graphic symbols they can understand, accessing new meaning systems (Piaget, 1984; DeLoach, 2011). In this way, the children understand that reality can be represented graphically, which will therefore prepare them for later use of conventional complex graphic symbols, such as writing, reading, and calculation, the main tools of cognitive and cultural development. For example, if there is a need for apples during play and there are no material substitutes available, the children can produce a graphic substitute which still holds the function of apples as an object of activity during play.

The qualitative analysis for the final assessment showed that children's drawings, after receiving the game program, achieved the parameters of the expected drawing. The graphic executions of the children matched the instructions given by the evaluator, which indicated that participants maintained the requested objective. The drawings were better organized and represented objects with essential details to be identified, such as the graphic sign “arrow.” For the children, drawing an arrow is a special means of fixing what is most important in the drawing (Liublinskaia, 1971).

This is how drawings in childhood are part of a reality that contains visual, tactile, and kinesthetic aspects, that is, action is form and form is action (Ruíz and Abad, 2011). Drawing as a dynamic interaction between the actions, intentions, and emotions of the child is essential for the formation of signs and symbols (Salmina, 2010). It has been referred that the understanding and use of images is the product of a slow and complex process where cognitive, social, and educational aspects converge (Salsa and Peralta, 2009), so that preschool education regains great relevance in child psychological development.

Another aspect to highlight is that in the final assessment, children managed to name and explain their drawings with coherent and complete verbal expressions. Vigotsky (1995b,c) points out that language becomes a tool in play because it allows children to share real and imagined meanings. During the game, children actively use language to coordinate, negotiate roles, rules, times, situations, and the conclusion of the game (Bonilla-Sánchez and Solovieva, 2016). Therefore, when interacting with other children in different game contexts, with different tasks and objects, the child restructures the personal meaning of words, gradually adopting the conventional meaning of the culture (Bodrova and Leong, 2003), also developing the reflective thinking (González et al., 2011). In this way, children benefit from the accumulated knowledge of previous generations (Cox M., 2005).

Our findings show that, in addition to the improvement of the drawings from the technical aspect, substantial changes in the sense and meaning expressed graphically and verbally by the child are clearly identified (Gross and Hayne, 1999). This indicates a cognitive advance, because the more complex the use and handling of symbols, the more complex the intellectual activity will be.

Piaget (1984) affirms that language is the vehicle of concepts that reinforces individual thought, because it reconstructs and evokes the past in the absence of objects until it replaces them with words.

From the perspective of linguistics, it has been referred that the drawings are part of the formation of semantic descriptions of the child's reality. Drawings are non-arbitrary symbolic representations, since there are rudimentary links between the signifier and the signified (Saussure, 2006). In this way, children's drawings are a form of language that contains meanings and, in turn, verbal language uses sounds and words as another symbolic form of representation (Cox S., 2005) and as a way of specifying the meaning of the drawing.

Finally, our results showed that was a statistically significant change in the category Intention or Reason to represent the idea or content. In the final assessment, children were able to represent the idea or content requested in the task's instruction. Therefore, the use of images, drawings, and maps as external media helped children to understand complex relationships (Bodrova and Leong, 2008).

The findings of this research concur with the perspective of other authors, confirming that social role play must be developed with the participation of adults, who guide the children in the actions to perform it (Talizina, 2009; González et al., 2011; Ruíz and Abad, 2011). It is also confirmed that the role play promotes the development of mental and social skills in preschool age, since children not only acquire appropriate information, but will also be able to reflect, re-create, develop new ideas, and create mental images and concepts, regulating their own emotions efficiently (Elkonin, 1980; García et al., 2013; González et al., 2014), facilitating the separation of thought from actions and objects (Bodrova and Leong, 2008).

Other benefits of using drawings are that children remember more easily and accurately the details about their experiences, structuring their memory progressively and sequentially, because this creative drawing skill provides them guidance on the conditions under which children can retrieve and express their memories in preschool age (Butler et al., 1995; Davison and Thomas, 2001; Barlow et al., 2011; Gardner et al., 2020).

Other authors have dedicated their efforts to identify the child's ability to understand and use symbolic representations from an early age (DeLoach, 2011). However, the way to promote the use and creation of symbols and signs on practical and motivating activities for children has not been indicated.

Previous research on psychology had used children's drawings, but with different purposes than those exposed in this article. A specific program had been proposed for the formation of drawing in the face of learning problems in writing, and the program had corrective purposes in a single case study of a school-age child (Mata et al., 2014). Some studies have used drawing as a facilitator of the psychotherapeutic process of children with autism (Gómez et al., 2012), and others have studied the evolutionary stages of children's drawing, trying to encourage the expression and creativity of children (Jolley et al., 2004; Jolley, 2010; Rojas, 2012).

In general, children's drawings have been used as the basis for assessing their intelligence or emotional stability, but psychologists and education specialists should use them as a test for identifying children's educational needs (Cox M., 2005).

### Limitations of the study

It is considered that the findings obtained cannot be generalized to the entire child population of preschool age. It is essential to consider the living conditions of minors, or rather, their social situation of development (Vigotsky, 1995a), as well as the way preschool education centers work, the possibilities of management and time for carrying out the games, and the availability of specialists to collaborate and coordinate such activity.

### Practical and theoretical implications

The use of role play based on the Historical–Cultural perspective can contribute toward the implementation of a ludic methodology in the educational field (Bodrova and Leong, 2003; Bonilla et al., 2012; González et al., 2014), constituting an alternative of psychopedagogical work that guarantees the psychological development and the expected learning in initial education. The practical implications of our findings support the inclusion of signs and symbols in children's games from an early age, guaranteeing positive effects on the development and content of drawing (Vigotsky, 1995a, 1996). Our findings confirm that role play is the activity that enhances development (Medina, 2010), an activity that intensifies human cognitive processing (Flavell, 2019), a social practice where the zone of proximal development of the child and the development of new adaptive behaviors in childhood are created (Rogoff, 1990).

Therefore, our study proposes that the school environment should offer teaching methodologies where the child is involved with artistic methods, such as drawing (Iordanou et al., 2022).

## Conclusion

Because role play is the guiding activity of preschool age, it guides the development of the symbolic function, where it is possible to apply the concept of the child's zone of proximal development with the guidance and help of adults and/or educator. It is possible to guide the development of children's drawing as a planned, organized, and thoughtful activity, gradually introducing preschool children to new meaning systems. This is an essential moment for the formation and mastery of signs and symbols on the graphic plane, preparing children to learn, read, write, and calculate.

The active use of language by children during the actions of the role play promoted the interaction between the playmates, making it possible for them to constantly restructure the personal and social meaning of the words, gradually enriching their verbal expressions or stories of their drawings.

## Data availability statement

The datasets presented in this article are not readily available because the data are confidential. Requests to access the datasets should be directed to MB-S, maria.bonilla@correo.buap.mx.

## Ethics statement

The studies involving human participants were reviewed and approved by Comité de ética local de la Maestría en Diagnóstico y Rehabilitación Neuropsicológica. Benemérita Universidad Autónoma de Puebla. Written informed consent to participate in this study was provided by the participants' legal guardian/next of kin.

## Author contributions

MB-S: proposal of the research topic and its design, data collection, qualitative analysis of the data, interpretation, and writing of the manuscript, critical review of the manuscript, and approval of the final version for publication. MG-F and IM-B: statistical analysis of data, interpretation of results, writing of the manuscript, critical review, and final approval of the manuscript. JS-G: data collection, processing and interpretation, and writing of the manuscript. ER-A: interpretation, writing, and critical review of the manuscript. All authors reviewed and approved the final version of the manuscript.

## Funding

This work was supported by Vicerrectoría de Investigación y Estudios de Postgrado de la Benemérita Universidad Autónoma de Puebla: VIEP-BUAP BOSMdR-EDH-22 (MB-S), Mexico.

## Conflict of interest

The authors declare that the research was conducted in the absence of any commercial or financial relationships that could be construed as a potential conflict of interest.

## Publisher's note

All claims expressed in this article are solely those of the authors and do not necessarily represent those of their affiliated organizations, or those of the publisher, the editors and the reviewers. Any product that may be evaluated in this article, or claim that may be made by its manufacturer, is not guaranteed or endorsed by the publisher.
